# Electrocharging face masks with corona discharge treatment

**DOI:** 10.1098/rspa.2021.0062

**Published:** 2021-07

**Authors:** M. M. Bandi, N. Ishizu, H.-B. Kang

**Affiliations:** ^1^ Nonlinear and Non-equilibrium Physics Unit, OIST Graduate University, Onna 904 0495, Japan; ^2^ Engineering Support Section, OIST Graduate University, Onna 904 0495, Japan

**Keywords:** electrocharged, respirator, face masks, cloth fabrics

## Abstract

We detail an experimental method to electrocharge N95 facepiece respirators and face masks (FMs) made from a variety of fabrics (including non-woven polymer and knitted cloth) using corona discharge treatment (CDT). We present practical designs to construct a CDT system from commonly available parts and detail calibrations performed on different fabrics to study their electrocharging characteristics. After confirming the post-CDT structural integrity of fabrics, measurements showed that all non-woven polymer electret and only some knitted cloth fabrics are capable of charge retention. Whereas polymeric fabrics follow the well-known isothermal charging route, ion adsorption causes electrocharging in knitted cloth fabrics. Filtration tests demonstrate improved steady filtration efficiency in non-woven polymer electret filters. On the other hand, knitted cloth fabric filters capable of charge retention start with improved filtration efficiency which decays in time over up to 7 h depending on the fabric type, with filtration efficiency tracking the electric discharge. A rapid recharge for a few seconds ensures FM reuse over multiple cycles without degradation.

## 1. Introduction

The COVID-19 pandemic has resulted in large-scale demand for N95 facepiece respirators (FRs) among essential personnel [1,2] and face masks (FMs) within the larger populace [3]. Supply chain disruptions in the pandemic’s early days necessitated innovative methods to decontaminate and reuse personal protective equipment (PPE) [4–11]. This was especially true for single-use non-woven polymer-based FRs and surgical FMs, in turn causing widespread adoption of wash-and-reuse cloth-based FMs [3] for non-essential personnel.

Following this first line of response to decontaminate and reuse PPE, several teams pivoted their research at short notice to address the myriad issues concerning PPE to keep up with evolving public health advisories [12]. In particular, innovative fabrication methods, designs and materials for FRs and FMs were proposed, including nanoparticle-loaded filters [13,14], affordable high-quality silicone elastomer [15] and three-dimensional printed FRs [16], electrocharged filtration layers manufactured using simple locally sourced components [16], etc.; Ahmed *et al.* [17] and references therein provide a detailed review.

However, non-woven polymer electret fabrics form the mainstay of reliable filtration, especially in N95 FRs, where they achieve high filtration efficiency through electrocharging [18,19]. Most surgical, non-woven, polymer-based FMs and standard cloth-based FMs do not possess this electrocharged filtration capability. Even in the case of N95 FRs, repeated decontamination and reuse degrades the embedded electric charges, resulting in reduced filtration efficiencies [20]. These considerations highlight the need for methods to charge FMs or recharge FRs in a rapid and reliable manner. Accordingly, various methods such as triboelectric charging of FMs [21–23] and exposing N95 FRs to high-voltage electric fields [20] have been reported.

In this article, we present an experimental study to explore charging in a wide variety of fabrics (both natural and synthetic) using corona discharge from the dielectric breakdown of air at atmospheric pressures, henceforth referred to as corona discharge treatment (CDT). Whereas CDT and its close relative plasma treatment are used to treat knitted or woven cloth fabrics to improve dye retention, flame resistance, etc. [24–30], applying CDT to electrocharge knitted cloth fabrics has not been reported to our knowledge. On the other hand, non-woven polymer electret fabrics are routinely treated with corona discharge to improve their electrocharging characteristics [31,32]. Although plasma, which is closely related to CDT, has been used to decontaminate N95 FRs [6], it does not recharge them. In fact plasma decontamination uses balanced ion generation, which bleeds existing charge from filtration layers, whereas electrocharging requires anion generation that can be achieved with negative unipolar CDT [16].

We first present designs to construct a CDT system that is capable of overcoming global logistics disruptions, as occurred during the pandemic, from common parts, e.g. a microwave oven or commercial neon sign power supply (NSPS). We discuss the relative design pros and cons and provide a detailed treatment protocol. Experimental measurements of structural characterization, charge retention and filtration efficiency of the fabrics pre- and post-CDT are described. We show that CDT electrocharges non-woven polymeric fabrics through the well-known isothermal charging process [16]. Despite a lack of prior results on CDT’s ability to electrocharge knitted cloth fabrics, a knowledge of triboelectric charging [33] formed our preliminary motivation to explore charging of knitted cloth fabrics. After investigating the operational parameters over wide ranges, we found that some knitted cloth fabrics can retain negative charges to varying degrees. We show experimentally how knitted cloth fabrics are electrocharged via ion adsorption, as opposed to isothermal charging in polymeric electrets. Unfortunately the most commonly used cloth fabrics in FMs, namely cotton, linen or silk, do not exhibit charge retention, but some natural, e.g. cellulose and natural polyester, and synthetic, e.g. nylon, fabrics were able to take up charges. FRs or FMs reveal a measurable difference in filtration efficiency pre- and post-CDT, which we used to establish a correspondence between the magnitude of charge retained and improvement in filtration efficiency. Filtration efficiency does progressively decay in knitted cloth fabrics over a duration of up to 7 h on account of electrical discharging, which varies with fabric type, but this presents no problem as CDT quickly recharges fabrics within a few seconds.

## 2. Background

### (a) Electrocharged filtration

Filtration presents a multi-scale problem ranging from hundreds of nanometres at the aerosol droplet or particle scale through a few to tens of micrometres for mask fibre diameters up to about a few to 10 cm dimensions at which masks cover the face. Like other multi-scale problems in physics and mechanics, filtration also currently lacks a tractable theory. Empirical and phenomenological results inform FR and FM design in achieving efficient filtration while retaining easy respiration [34] through a set of three basic filtration mechanisms [35]. The first of them is inertial impaction, whereby large particles of diameter ≥1 μm deviate from flow streamlines as a result of inertia and impact the filter mesh and become trapped. The second mechanism applies to small particles of diameters ≤0.1 μm, which diffuse while following flow streamlines within the porous fabric and come to rest on its fibres.

The third mechanism of electrocharged filtration [18,19] applies in the intermediate range of diameters, i.e. 0.1–1 μm, where neither inertial impaction nor diffusion are effective. Here, natural or embedded dipoles in the filtration fabric set up a static electric field around the mask layers to attract charged dust particles and aerosol droplets and trap them through weak van der Waals forces [35]. Particularly in the case of N95 FRs and their equivalent counterparts in other countries, the filtration standard requires such FRs to filter out 95% of particles of diameter 3 μm or smaller, and electrocharged filtration, together with other design features such as tight facial fit and a gap between the face and inner mask surface to improve respiration, help to achieve this stringent filtration requirement.

Inertial impaction and diffusive filtration mechanisms are easily achieved with any fabric, including knitted cloth. The regularity of knitted/woven threads in a grid does reduce the tortuosity of airflow streamlines, which lowers filtration efficiency, but this is easily overcome by using different knit patterns for each layer and intentionally misaligning their grids between layers. However, electrocharged filtration has thus far been limited to electret polymer fabrics, with limited success from triboelectrically charged knitted or woven cloth fabrics. An easy and universal method to electrocharge any arbitrary fabric, be it natural or synthetic, knitted/woven cloth or non-woven, is desirable.

### (b) Corona discharge treatment

Air is normally a good insulator with electrically neutral molecules. When imparted with a high magnitude of energy in the form of electrons streaming from an electrode colliding with the molecules, the electrons orbiting the atomic nuclei absorb the energy and shift to higher energy levels and eventually dissociate from their constituent atoms or molecules, leaving behind a positively charged ion and the dissociated free electron. This avalanche process, whereby new electrons are produced by incident ones, results in a mixture of electrons and ions.

**Figure 1.**
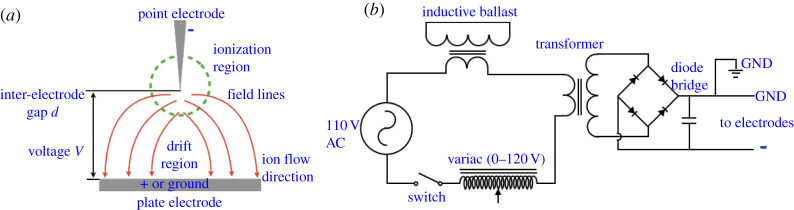
(*a*) Basic schematic for a point-plate electrode CDT system showing the ionization and drift regions and field lines in the direction of ion flow. (*b*) Schematic of the basic electric circuit for a CDT system. GND, ground. (Online version in colour.)

A corona discharge occurs when air or any gas is subjected to ionization within a region of space exposed to high electric fields using electrodes. Depending on the electrode polarity the corona can be unipolar (either positive or negative) or biopolar, AC or DC, or even high frequency [36]. Whereas some characteristics of corona discharge vary with polarity type, they all have a region where electrons collide with neutral molecules to set off ionization, followed by a drift region where these ions and electrons drift towards electrodes of opposing polarity (see figure 1*a* for a schematic). Electrons with high energy collide with neutral molecules to dissociate them into electrons and ions within the ionization region [37] (figure 1*a*). If they lose energy post collision, new collisions with neutral molecules in the drift region do not induce further ionization and the corona polarity can be maintained. If sufficiently high energy remains post ionization, these electrons and ions can react with other ionized particles, resulting in plasma. Although the terms CDT and plasma are interchangeably used, since the current work focuses exclusively on unipolar discharge of negative polarity, we use the term CDT in this study to avoid any confusion.

Both corona and plasma discharge find use in surface treatments in industry and research [38]. Glass and plastic containers are passed under such discharges for decontamination in industrial packaging. Plasma treatment is similarly used for surface decontamination and reuse of PPE in healthcare settings [6]. Plasma and CDT are employed in laboratories for removal of trace organic impurities from glass [39,40], especially in surface measurements [41], or to treat silicone elastomers for bonding [42]. Perhaps the best-known discharges in familiar settings are fluorescent lights and neon signs, and indeed, as we show later, the simplest design for construction of a CDT system is with an off-the-shelf NSPS. Finally, as mentioned previously, surface treatment of knitted/woven cloth with plasma [24–30] to induce surface reactions that render the cloth fire resistant, wrinkle free or with improved dye retention properties have been extensively explored.

The primary goal of the present work is to exploit the textile industry’s plasma treatment methods and minimally modify them to achieve corona treatment and impart surface charges onto arbitrary fabrics. We wish to achieve this with a rapid, reliable and controlled protocol, so that FMs can be washed, electrocharged via CDT and reused several times without any degradation in structure and/or filtration efficiency. Finally, we want to achieve all of these goals using off-the-shelf components that are easy to source locally with a simple and safe design.

## 3. Corona discharge treatment system design

We first outline the control parameters for the design of a CDT system’s electric circuit and further simplify it for construction with off-the-shelf components. Design modifications at the electrode end and the electrocharging method that diverge from standard textile processing using CDT are then explained.

### (a) Control parameters

Both plasma and CDT require the application of a high-voltage electric field through a gas using electrodes which produce highly energetic electrons or ions. These electrons or ions then dissociate electrons from the gas molecules to produce ions in a region close to the active electrode, usually called the ionization region. The electrons and ions then respond to the electric field and drift towards electrodes of opposing polarity; this region away from the active electrodes is called the drift region. These mobile ions provide a dynamically varying percolating path for the mobile electrons to traverse across the electrodes to complete the circuit, observed in the form of a spark or arc, called streamers. Two primary control parameters inform the design of such a system, namely the threshold electric field magnitude necessary to cause ionization and the voltage–current characteristic that achieves steady-state ionization and drift. The lower bound is set by the breakdown voltage (*V*_*B*_), the minimum voltage at which the dielectric breakdown of air is achieved and an arc of plasma discharge is initiated across an electrode gap. Although no theoretical upper bound exists for the voltage, there is an upper bound for the current, which saturates at a specified voltage, called the saturation current (*I*_*S*_). A third parameter is crucial to corona discharge but not for ionization onset from breakdown. This is the corona inception voltage (CIV) at which a visual corona discharge occurs. The CIV is not important for our purposes as the primary focus of this work concerns ion generation; CIV becomes important when suppression of corona discharge is desired, e.g. high-voltage lines. For informational purposes, CIV is determined empirically by Peek’s law and its refinements; the interested reader is directed to Peek [43] and Kuffel *et al*. [44] for further information.

#### (i) Breakdown voltage

For a specified gas, the breakdown voltage is a function only of the product of the pressure and gap distance between the electrodes, and is governed by Paschen’s law [45]:

3.1 $$V_{B}=\frac{Bpd}{\text{ln}\;(Apd)-\text{ln}[\text{ln}(1+(1/\gamma_{\text{se}}))]}.$$

Here *V*_*B*_ is the breakdown voltage in volts, *p* is the gas pressure in pascals, *d* is the gap distance between electrodes in metres, $\gamma_{\text{se}}$ is the secondary electron emission coefficient (the number of secondary electrons produced per incident ion in the avalanche process that initiates ionization), *A* is a constant representing the saturation ionization in the gas at a particular *E*/*p* (electric field *E* to pressure *p*) ratio and *B* is a second constant related to the excitation and ionization energies. The constants *A* and *B* are determined experimentally and are normally constant over a range of *E*/*p* values for a given gas. We note that equation (3.1) is not an exact result, but a good approximation that applies for a range of the product *pd* at higher pressures and inter-electrode gap distances. Whereas equation (3.1) was empirically determined by Paschen from experimental data in 1889 [45], its derivation was provided by Townsend [46].

Plotting equation (3.1) for *V*_*B*_ versus *pd* provides the constitutive Paschen curve for a specified gas. Differentiating *V*_*B*_ with respect to the product *pd* and equating the resultant derivative to zero yields

3.2 $$pd=\frac{e\cdot \text{ln}\;(1+(1/\gamma_{\text{se}}))}{A},$$

from which the minimal breakdown voltage is discerned. Keeping pressure *p* fixed at 1 atm (atmospheric pressure), as done in the present study, the composition of the gas then determines the minimum *V*_*B*_ and the minimal inter-electrode gap *d* at which dielectric breakdown occurs. For instance, at atmospheric pressure (p=1 atm=101.325 kPa), air has $V_{B}=327\;\text{V}$ at d=7.5 μm [47]. Since practical systems have *d* in the range of millimetres (e.g. spark plugs) to centimetres (e.g. textile treatment systems), a simple way to estimate *V*_*B*_ from equation (3.1) is to lump *A*, *B*, $\gamma_{\text{se}}$ into a single parameter. In this manner, one can arrive at a ballpark estimate by simply multiplying *d* in centimetres by a factor of 3 × 10^4^ to obtain $V_{B}∼3$–30 kV for d = 0.1–1 cm. This range of *V*_*B*_ informs our design of CDT circuitry, especially the transformer needed to step up the voltage fed from a standard 110 or 220 V power source.

#### (ii) Saturation current

Well above the breakdown voltage a stable, concentrated ionization region is set up near the active electrode and the space charge field determines the voltage–current relation of the corona discharge system. A lumped parameter analysis approaches the space charge field by treating the ionic drift region as a large, nonlinear resistor in series with the ionization region. Despite the simplicity afforded by a lumped parameter treatment, the resulting complex space–charge flow problem is captured by a nonlinear integrodifferential equation. Solutions to this equation are permissible either under extreme simplification that linearizes the problem or for special geometries possessing high symmetry. The steady-state spatial distribution of ion drift between electrodes, called the Warburg distribution [48,49], when spatially integrated yields an experimentally accessible relation between the voltage *V* and the saturation current *I*_*s*_ (amp) through Sigmond’s approximation [37],

3.3 $$I_{s}∼\frac{2\mu \varepsilon V^{2}}{d},$$

where *μ* is the unipolar ion mobility in ${\text{m}}^{2}\;{\text{V}}^{-1}\;{\text{s}}^{-1}$, and *ε* is the permittivity of the inter-electrode medium in F m^−1^. Sigmond’s approximation, which was originally derived for point-plate geometry, also extends to wire–cylinder and wire–plate geometries.

A few qualifications are in order before we proceed. Firstly, the nonlinear relation between voltage and current is immediately apparent from the fact that equation (3.3) exhibits a quadratic dependence despite the approximations arising from a lumped parameter analysis and simplification of the nonlinear integrodifferential equation. A comparison with Ohm’s law would be erroneous for it does not apply here; equation (3.3) is not concerned with the amount of current passing through a medium of given resistance at a given voltage. The resistance of the gas is variable as part of it undergoes ionization (conducting) and the rest does not (insulating) and both ions and molecules are mobile, unlike a solid-state current-carrying conductor. Equation (3.3) instead provides an estimate of the maximum (saturation) current that can pass through the medium at a given voltage. The quantities addressed by the linear Ohm’s law and Sigmond’s approximation are therefore unrelated. Secondly, in textile treatment processes the cloth sample is placed between the electrodes, implying that the inter-electrode medium comprises both air and a cloth dielectric, thereby necessitating a more nuanced treatment of *ε*. As discussed later, we deviate from this procedure by allowing us to use the value for air, which yields a simpler form of equation (3.3): $I_{s}∼(4V^{2})/d$.

Ionic flow in air-based corona discharge is collision dominated: nearly all ionic momentum and energy provided by the electric field is transferred to air’s neutral gas molecules. This momentum transfer renders the ionic flow viscous and causes a strong axial jet [50] known as electric wind, which is measurable [51] and which can be employed in the manipulation or extinction of flames [52]. As an order-of-magnitude estimate, CDT using air at atmospheric pressure with 10 kV applied voltage yields a saturation current in the tens of micro-amps range and the electric wind flows with a velocity of a few metres per second. If the CDT has positive polarity, nearly 95% of the input field energy is transferred to the neutral gas in the form of heat since positive ions can hardly excite neutral molecules in the drift region. However, in negative polarity CDT, a much higher current can pass through the gas because an increasing number of electrons can dissociate and escape neutral atoms, which, in turn, excite and ionize more neutral molecules, thereby extending the ionization region further out. For this reason, the electric wind for negative corona tends to have a slightly higher mean velocity than corona of positive polarity [51]—a fact that works in our favour, as explained in the electrode design subsection below.

### (b) Design

Having identified the control parameters and applied them to obtain an order-of-magnitude range of voltage and current values, we now translate them into a practical design and explain how it can be constructed from readily available components. We require a unipolar CDT system designed for negative polarity constructed using easily accessible parts and capable of charging a wide range of fabrics with possibly different underlying charge retention mechanisms.

#### (i) Basic circuit

Before we begin, CDT systems operate at high voltages in the few to tens of kilovolts range. We caution the reader to exercise all precautionary measures when working with them. The most basic rule that everyone should follow is to switch off the circuit and disconnect the cord from the AC power source before starting any work on the circuit. If using a capacitor for a DC CDT system, as we show below, you should wait of the order of 10 s, but it is advisable to wait up to a minute for the capacitor to completely discharge after disconnecting the power source before approaching the circuit. Only power cords rated for the power capacity should be employed; this is easily achieved by using cords and components with the standard electrical safety certification symbols such as VDE (European Union), CE (European Union), UL (C-UL (Canada), C-UL USA (Canada and USA)) and ETL (USA and Canada). Several of these standards are universally accepted. The reader is strongly advised to look up the standard electrical safety certification symbols for their local region before constructing their CDT system.

The basic electrical circuit for a CDT system shown in figure 1*b* draws its input power from a standard 110–220 V AC wall-mounted power source capable of supplying up to 10 A of current. Moving counterclockwise from the 110 V AC source one could use a rating-certified extension cord, leaving the end that plugs into a wall-mounted power socket intact, and cut the other end of the extension cord to reveal the bare wiring. After stripping the hot (also known as live) and neutral wires and ignoring the ground (also known as earth) wire, should the extension cord have one, either the hot or neutral wire is connected to a safety switch in series to one side of the primary of a step-up transformer capable of raising the voltage by a few 10× multiple. The easiest sources for such transformers in commonly available appliances are microwave ovens, where they are usually called MOTs or microwave oven transformers. MOTs normally step up voltage 20× to an effective voltage of 2 kV and effective current of 0.5 A, generating a peak effective power of up to 1000 W. For the purposes of generating tens of kilovolts, one can set up two MOTs in series, obtaining the requisite boost in voltage. Note that wiring the outputs of MOTs or any step-up transformers in series adds up voltage at constant current, whereas wiring them in parallel adds up current at constant voltage; since we seek to increase the voltage, we wire the outputs in series. Wiring the primary end of the transformer is usually straightforward, but if one is not a trained electrician or lacks experience with transformers wiring the secondary part of the transformer can be confusing. If the MOT or commercial transformer has two output wires at the secondary end then the wiring is straightforward, but some models occasionally have one side of the secondary soldered to the main body of the transformer itself. In such a situation, soldering another wire to the end soldered to the transformer’s main body suffices, but it should strictly be done while the entire circuit is powered down and disconnected from the electrical mains.

**Figure 2.**
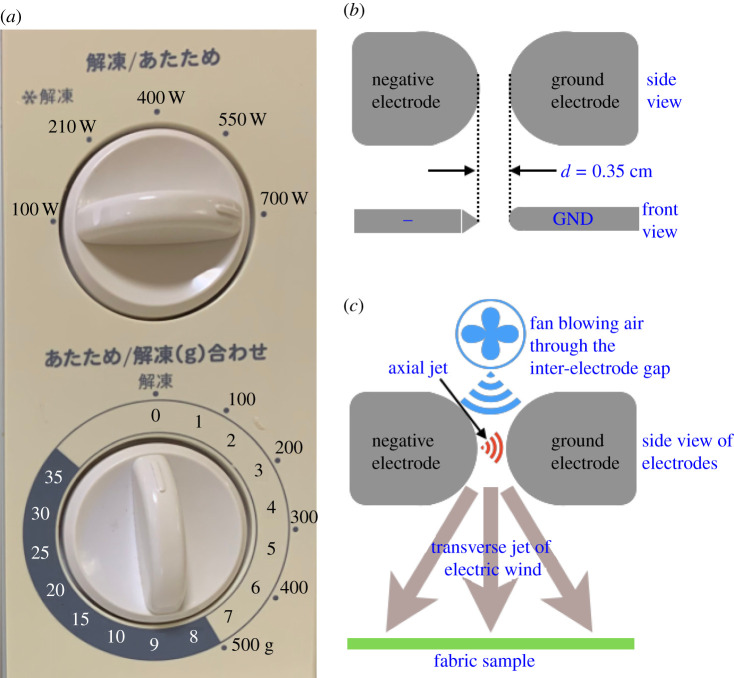
(*a*) The knob for microwave power is a discrete variac that regulates voltage at constant current. (*b*) Side and top views of the electrode design for the CDT system shows two flat plates with a semi-circular geometry along the direction facing each other. The ion-emitting negative electrode has a sharp tapered edge whereas the ground electrode has a rounded edge to mimic the point-cylinder geometry with an inter-electrode gap d=0.35 cm. (*c*) A centrifugal fan blows air through the inter-electrode gap to deflect the axial jet traversing from the negative to the ground electrode and converts it into a transverse jet of electric wind that transports the ions onto the fabric. (Online version in colour.)

Connecting the two wires from the secondary to the electrode plates is sufficient to obtain a basic CDT system that simply steps up the output voltage and generates corona discharge. However, this circuit operates in a simple on-and-off mode with absolutely no voltage or current control. The addition of a variac in series with the switch and one side of the MOT’s primary achieves one part of the desired control. The variac helps to control the input voltage into the transformer’s primary, usually in the range 0–120 V. Having the variac before the primary input helps to control the effective peak output voltage at the transformer’s secondary end by allowing a gradual voltage rise from zero across *V*_*B*_ until the saturation current is achieved. The variac also helps in experimental calibrations of the optimal output voltage, depending on the fabric sample chosen. Finally, the variac is also a secondary safety feature that helps to bring down the voltage to safe levels in case the circuit does not work as expected. The variac is perhaps the only component that is not readily available in everyday electrical appliances. However, many models of microwave ovens come with adjustable power in discrete steps (see figure 2*a* for an exemplar), which in reality is a discrete step variac that regulates power by regulating voltage at constant current. The only difference is that a commercial variac permits continuous smooth variation in input voltage whereas a repurposed variac from a microwave offers the variation in a few discrete steps. Such discrete-step regulators from microwaves suffice for a CDT system if a commercial variac is unavailable.

Having accomplished voltage control that helps us get past *V*_*B*_ (equation (3.1)), we can now connect an inductive ballast between the second wire coming off the power source to the second wire of the transformer’s primary. The inductive ballast is a current-limiting component that limits the current flowing across the circuit and serves two purposes. Firstly, it allows the circuit to reach saturation current *I*_*S*_ for any voltage value $V\geq V_{B}$ without the need for manual current control, thus obviating the need for equation (3.3). Secondly, it serves as a safety feature by preventing the circuit from drawing too much current and potentially blowing up the circuit and endangering personnel. The inductive ballast is just another transformer (a second MOT serves quite well) with the secondary shorted, i.e. both terminals of the secondary are wired to each other, and placed in series with the primary of the main transformer. As shown in the circuit presented in figure 1*b* one end of the inductive ballast is connected to the power source and the other end to one end of the main transformer’s primary. This completes all details for the input side of our CDT circuit.

On the output end of the circuit, we have the two terminals emerging from the secondary of the main transformer. Since we started with an AC power input, we referred to the two wires as hot and neutral, rather than positive and negative, since AC supplies cycle periodically between positive and negative at 50 or 60 Hz depending on the country. The circuit described thus far is sufficient to generate corona discharge in AC mode. Now we go a step further and include a full-wave bridge rectifier, also known as a diode bridge, to convert AC to DC voltage (figure 1*b*). The simplest way to build the diode bridge is by using the high-voltage diodes that come with microwave ovens; the diode bridge is usually encased with the capacitor near the magnetron system and the diodes are rated usually for 12 kV at 350 mA. Adding a capacitor, also encased with the diode bridge in a microwave oven, and connecting in parallel with the diode bridge as shown in figure 1*b* completes the circuit. The two output terminals from the diode bridge (figure 1*b*) then connect to electrodes to complete the CDT circuit.

Grounding the positive output terminal before it terminates in an electrode renders the CDT system unipolar with negative polarity; the grounded electrode is now passive and the CDT system is essentially an anion generator. If the positive terminal is not grounded, one obtains a balanced bipolar CDT system known as an electrostatic discharge ionizer or balanced ion generator, normally used to generate ozone to decontaminate surfaces. This simple design can therefore serve a dual purpose as an anion generator when the positive terminal is grounded to electrocharge PPE and as a surface decontamination system if the positive terminal is left ungrounded.

An even simpler and more importantly safer design replaces the inductive ballast and main transformer with an NSPS, with the remaining secondary components, namely the switch, variac, diode bridge and capacitor, connected to the NSPS. Our tests yielded identical results when we replaced the inductive ballast and main transformer in figure 1*b* with an Allanson SS1235ICH NSPS (peak effective voltage: 2–12 kV, peak current: 0.35 mA). The NSPS-based CDT system encases the high-voltage ballast and transformer, providing relatively more operational safety to a novice designer.

The CDT design we used for the present study employed two 6.7 kV main transformers in series (Tokyo Transformer Co. Ltd; model 17124B) providing a 13.4 kV output, a regular MOT salvaged from a discarded microwave oven for the inductive ballast and a commercial variac (Beleeb Inc.; model no. TDGC-0.5KM; operating voltage: 0–120 V, operating current: 5A). With a peak transformer voltage of 13.4 kV, we could not use salvaged high-voltage microwave diodes as they were rated only up to 12 kV. Instead, we used commercial high-voltage diodes (Dean Technology; 2CL2FM; rated for 20 kV peak voltage and 100 mA peak forward current and 2 μA peak reverse current) and a high-voltage capacitor (General Electric; model no. WB27X10701; 1.05 μF) from a discarded microwave oven. The circuit was encased in a box to ensure safe operation and the output leads were drawn from the box in a PVC tube terminating in the electrode. Doing so allowed us to place the circuit with its heavy transformers at a stationary location and mount the electrodes on a simple linear guide rail system (see electronic supplementary material, movie M3.mov), allowing us to expose the fabric to the output transverse jet of electric wind in a raster scan (see electronic supplementary material, movie M4.mov showing the CDT treatment of a surgical mask (SM)).

### (c) Electrode design

The design thus far is unrelated to the type of application, except for the choice of a unipolar (negative or positive) versus bipolar corona discharge system. As mentioned earlier, electrocharged filtration application requires unipolar negative CDT (anion generator), but with minimal modification by ungrounding the positive electrode this same system becomes an ozone generator for decontamination of PPE [6]. Whereas the present study does not explore PPE decontamination with this CDT system run in plasma mode, we inform the interested reader of this possibility. A simple protocol would be to run the CDT in bipolar mode to decontaminate FRs and FMs followed by a second CDT run in negative unipolar mode to electrocharge the samples.

The deviation from the textile-processing applications we mentioned earlier commences with the design of the electrodes, which form the interface between the CDT system and the sample to be processed. Typical textile plasma treatment systems place the sample between the electrodes, whereby the air surrounding the active electrode breaks down to generate the desired ions, which then drift through the cloth sample in an attempt to reach the electrode of opposing polarity; in doing so, they perform the desired treatment on the cloth sample’s surface.

Given that our goal is to embed negative charges in the fabric and not perform a permanent treatment, we follow a different design for two reasons. Firstly, unipolar negative corona systems with point-plate geometry initially generate a pulsed corona known as Trichel pulses [51], followed by a pulseless corona and eventually terminating with a spark discharge with increasing voltage. In wire–cylinder and wire–plate geometries, the unipolar negative corona is observed as a rapidly moving glow with concentrated active spots, usually called tufts or beads [53]. This implies that there is no escape from high-energy sparks, which, in the process of reaching the opposite electrode, intrude into the fabric sample and damage it with small pinhole burns. Secondly, we want to apply the minimal voltage necessary to achieve steady-state corona for safety, which in turn translates to a requirement for the least possible inter-electrode gap; see equations (3.1) and (3.2). However, at the same time the inter-electrode gap (*d*) must be large enough to permit a drift region and an electric wind.

**Figure 3.**
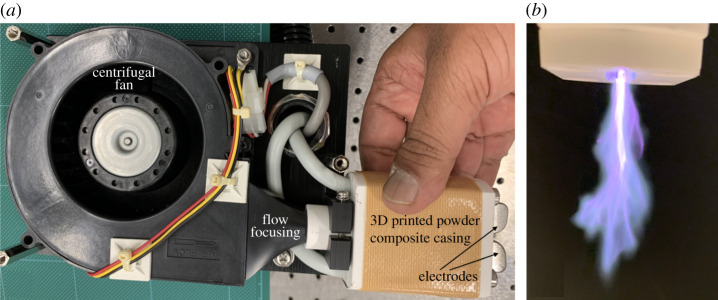
(*a*) Electrode system showing the centrifugal fan with a flow-focusing nozzle whose tapered conic section terminates at one (left) end of a three-dimensional printed casing made from heat-insulating powder composite; the casing houses the electrodes at the other (right) end. (*b*) Snapshot of the corona discharge’s transverse jet of electric wind exiting from the electrode end of the three-dimensional printed powder composite casing. (Online version in colour.)

With these practical considerations in mind, we designed electrodes that mimic the wire–cylinder geometry with a curvature as shown in figure 2*b*. The two electrodes were cut from tool steel of 1.5 mm thickness in a semicircular geometry as shown in figure 2*b* (side view), but one of them (negative electrode) was filed to have a sharp tapered edge while the second electrode (ground) had a smooth semicircular edge, thereby creating a design analogous to a wire–cylinder geometry. Tool steel was employed as this family of carbon and alloy steels is known for its hardness, wear resistance, toughness and resistance to softening at the elevated temperatures that arise at the electrodes, which can cause sputtering [54] and corrosion [55]. The inter-electrode gap was set to d=0.35 cm at the nearest point between the electrodes where the field intensity would be maximum, thus translating to a requirement of approximately $V_{B}∼10.5\;\text{kV}$, well within our design range. Air was blown from behind the electrodes with a centrifugal fan to reorient the axial jet traversing from the negative to the ground electrode in the transverse direction (see the schematic in figure 2*c*). One can control the airflow rate by introducing a regulator for the centrifugal fan, but we did not implement this in the current study. The semicircular electrode geometry allowed the corona discharge to smoothly slide along the electrode surface and eject a strong transverse jet of electric wind as shown in figure 3*b*; also see the coronal discharge exiting from the electrodes in slow motion at 240 frames per second in electronic supplementary material, movie M2.mov, where the Trichel pulses can be heard as semi-periodic clicks, the sparks can be observed at the nearest inter-electrode gap and the concentrated active spots are seen as a flame-like glow because they diffuse and spread out under influence of the air blown from the centrifugal fan. The fabric samples were exposed to this transverse corona jet for electrocharging purposes.

In practice, the electrodes were mounted onto one end of a three-dimensional printed powder composite casing with a flow-focusing nozzle at the other end. The casing was 3D printed using a ProJet 460Plus from 3D Systems and a component infiltrate (Colorbond) was applied to provide additional strength to the casing, which was printed with powder composite (Visijet PXL Core) combined with a binding agent (Visijet PXL); the powder composite has high thermal and electrical insulation properties as opposed to standard acrylonitrile–butadiene–styrene thermoplastics, which deform and melt under the heat generated from corona discharge. If lacking access to a powder composite 3D printer, we suggest using Bakelite handles from domestic utensils and drilling holes in them to place electrodes. The nozzle focused the air exiting the outlet of the centrifugal blower fan (HK Fan; model no. BB5015H12; DC 12 V brushless, flow rate: 35 cfm or approx. 1000 l m${\;}^{-1}$), as shown in figure 3*a*. The focusing nozzle therefore increased the flow rate as it approached the electrodes. Finally, for safety reasons a 3D-printed powder composite plate with a tapered slit was mounted onto the casing to enclose the electrodes, such that the transverse jet of the corona discharge exited through the slit. Albeit highly unlikely, to avoid electrical contact from dielectric breakdown of the powder composite, four plastic (instead of metallic) screws were used to mount the powder composite plate onto the powder composite casing. The resulting transverse jet of the corona discharge was observed as a jet exiting the slit in the powder composite plate as shown in figure 3*b* (also see electronic supplementary material, movie M1.mov at 30 frames per second and M2.mov at 240 frames per second without the powder composite plate, thus exposing the electrodes).

An important design consideration was the use of commonly available parts that can be sourced locally. All parts employed in the CDT design above can be locally sourced in any part of the world. In particular, the parts are relatively inexpensive and the system can be built on a frugal budget. For instance, the cost of all parts in our CDT system was less than the Japanese yen equivalent of US$1000. As an example, the cost of parts for this system would be approximately US$270 in China and US$320 in India. Sourcing of 3D-printed components could pose a potential challenge in developing countries, but, as explained earlier, Bakelite sourced from home utensils is an affordable alternative.

## 4. Characterization

With the CDT system design in place, the basic treatment protocol is quite simple as long as due caution is exercised to avoid accidental burning or electrocution. The protocol involves placing the fabric on a clean, flat surface with the CDT system electrodes facing down onto the fabric’s top surface and turning on the CDT system and exposing the fabric to incident ions for a sufficient duration until the fabric is electrocharged. In practice, however, one must determine some operational parameters, such as optimal distance between the electrodes and fabric and the treatment duration. These operational parameters depend on the applied voltage and the centrifugal fan’s airflow rate, both of which determine how far the discharge sparks and glow extend as well as the mean ion flux from the electrodes to the sample. Since both voltage and the airflow mechanism vary with implementation, the characterization tests help us demonstrate the method’s efficacy while providing details of tests that users should perform on their design implementations.

### (a) Structure

Since our aim is to achieve repetitive electrocharging, it is necessary that CDT does not cause irreversible structural changes to the fabric. Whereas one form of structural change occurs from fabric degradation due to discharge sparks or heat, another occurs from the usual treatment in the textile industry which changes the nature of the bonds on fabric surfaces, both of which we wish to avoid. We show under what conditions such structural changes occur, how to avoid them and how to achieve the desired result. Since structural degradation in non-woven polymer fabrics is different from that in knitted cloth fabrics, we explain both situations separately.

#### (i) Non-woven polymer fabrics

FRs and SMs normally employ melt-blown polymers as filtration layers with and without electrocharging, respectively. Whereas only the middle layer in FRs tends to be electrocharged, the outer layers of FRs and all layers of SMs are capable of being electrocharged as well. This holds true for SMs only when they are fabricated from non-woven polymers. Such electrocharging capability is the result of the fact that polymeric materials like polypropylene and polystyrene are electrets, i.e. they are capable of holding static charge through induced or oriented dipoles, which is easily achieved post-fabrication by exposing the fabrics to either an external electric field or CDT, commonly referred to as isothermal charging [16]. Due care must be observed when using CDT not to expose fabrics to sparks or heat generated by the discharge as they will most certainly degrade the fabric structure.

**Figure 4.**
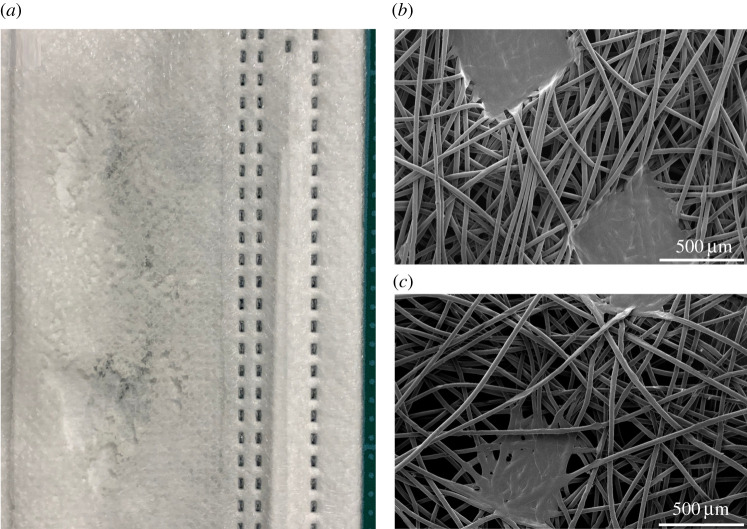
(*a*) Section of a post-DCT SM shows degradation from melting of the polymer when directly exposed to heat and sparks emanating from CDT electrodes. (*b*) Scanning electron micrograph of a pre-CDT commercial SM with high-quality polymer fibres. (*c*) The same SM post-CDT when the discharge sparks and flames were 3 cm away from the mask surface reveals degradation from local, microscopic melting or fraying of polymer fibres, thus rendering the mask unfit for use. Full resolution images are available in the electronic supplementary material. Full-resolution scanning electron micrographs are available in [56].

Isotactic polypropylene has a melting temperature of 160–165°C whereas polystyrene has a glass transition temperature of 100°C. As a result SMs readily melt if directly exposed to the high-temperature corona discharge, as demonstrated in a deliberate example in figure 4. For this reason, FRs and SMs should never undergo CDT in direct contact with discharge sparks. Even when the electrodes are distant enough from FRs and SMs such that the discharge does not come into direct contact with them, the transverse electric wind can be sufficiently hot if the distance is not large enough and this will result in SM degradation at the microscopic level through local melting or fraying of polymer strands. As an exemplar, figure 4 shows scanning electron micrographs for the same SM pre-CDT (figure 4*b*) and post-CDT (figure 4*c*) where the discharge sparks and glow are only 3 cm distant from the SM surface. These scanning electron micrographs were imaged by platinum–palladium sputter coating deposition of the fabric sample surface for visualization, followed by interrogation under a Quanta 250 FEG (FEI Thermo Fisher) scanning electron microscope at 2 kV acceleration voltage.

**Figure 5.**
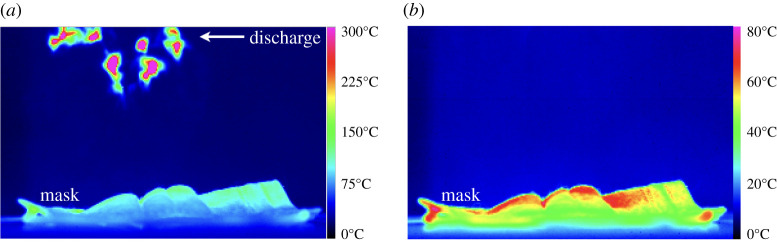
Heat map of the temperature field in the vicinity of the CDT system and SM when the discharge sparks and glow are (*a*) 3 cm and (*b*) 7 cm from the SM surface. Note that the colour spectra used to render the heat maps, albeit the same in both images, represent different temperature ranges. (Online version in colour.)

Avoiding heat-induced degradation of FRs and SMs by placing the electrodes sufficiently far from the mask surface also reduces the ion flux incident on the mask owing to divergence of the transverse electric wind. An optimal distance therefore exists at which the masks no longer degrade under heating while receiving sufficient ion flux. This was empirically determined by measuring the temperature field in the vicinity of SMs undergoing CDT using a medium wave infrared (MWIR) camera (FLIR Systems Inc.; model SC7000; image resolution: 320 × 256 pixels at 383 frames per second with a 25 mm 1:2.0 focal length lens and temperature range: 3–300°C). Figure 5 shows heat maps obtained from instantaneous snapshots for the same SM under identical conditions, except for the vertical distances between the SM and the discharge spark and glow of 3 cm (figure 5*a*) and 7 cm (figure 5*b*). Note that the heat map’s colour spectral range is identical between figure 5*a* and 5*b*, but they represent different temperature ranges.

An SM undergoing CDT at the vertical distance between mask and discharge glow of 3 cm (electronic supplementary material, movie SMCDT-3cm.mov) and 7 cm (electronic supplementary material, movie SMask-7cm-CDT.mov) does not show any visible differences between the two situations. However, the same two situations when interrogated with an MWIR camera clearly show that the SM attains mask surface temperatures in the range 100–110°C for the 3 cm vertical gap (figure 5*a* and electronic supplementary material, movie SMIR-3cm.mov) compared with mask surface temperatures of approx. 65–75°C for a 7 cm vertical gap (figure 5*b* and electronic supplementary material, movie SMIR-7cm.mov). A surface temperature of 100–110°C with a 3 cm vertical gap is sufficient to locally melt the fabric. We also draw the reader’s attention to the fact that the transverse electric wind can be observed as minor temperature fluctuations in the spatial region between the mask and the discharge in the thermal imaging movies presented in the electronic supplementary material.

In the aforementioned thermal imaging of the CDT process, we presented the limiting cases. In reality, the tests were performed for a range of vertical distances of 3–10 cm between the mask surface and the discharge glow in steps of 1 cm. The vertical distance of 7 cm was empirically determined to be the minimum distance, but we set the vertical distance at 8 cm for treatment of non-woven polymeric fabrics.

#### (ii) Knitted cloth fabrics

FMs made of knitted cloth, be they natural or synthetic, also undergo structural degradation under direct discharge contact, but for reasons different from those for non-woven polymeric fabrics. Knitted cloth fabrics tend to locally burn by singeing threads rather than through melting. This is observed as a spontaneous yellow flash from a burning thread and is spectrally distinct from the blue glow of the corona discharge (see electronic supplementary material, movie CMask-CDTburn.mov for an exemplar). These singes cause microscopic damage by burning individual threads that loosen the knit and consequently increase the pore diameter that is so essential to filtration efficiency.

**Figure 6.**
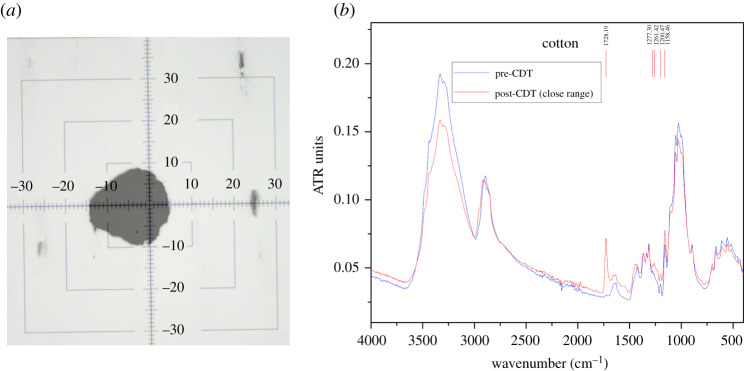
(*a*) Micrograph of a microscopic pinhole burn (dark patch) in cotton fabric (grey background) obtained under 4× magnification. A calibration cross-hair with gradation steps of 10 μm is superimposed on the micrograph.(*b*) FTIR results for a cotton fabric sample pre- and post-CDT at close range (3 cm). Data for this figure are available at [56]. (Online version in colour.)

A second form of structural degradation occurs in knitted cloth FMs when discharge sparks impart pinhole burns of micrometre dimensions in the mask fabric, thus rendering the FM completely unsuitable for use. Figure 6*a* shows a micrograph of a pinhole burn (black spot) in a cotton fabric (grey background) observed under a standard microscope at 4× magnification. An image of a calibration cross-hair with gradations in 10 μm steps is superimposed on the micrograph to demonstrate that the pinhole burn is approximately 10 μm in size. Given that most viruses and aerosol droplets are smaller than 10 μm—for instance the SARS-CoV-2 virion is estimated to be of the order of 50–200 nm in diameter [16,57,58]—such pinhole burns severely compromise the filtration efficiency of knitted cloth FMs.

Finally, a third form of change occurs when the CDT discharge glow is at a distance of 1–3 cm from the knitted cloth FM surface. To call it a structural change would be a misnomer since it involves the production of functional groups on a cloth fabric surface when the active species, such as free radicals generated by the air ionization, react with atoms or molecules there. This process is often researched in textile surface treatment methods [24,59] and usually does not result in any perceptible structural changes in the fabric, but rather in their functional properties, such as non-wrinkling or fire retardation. These functional groups can be detected via Fourier transform infrared (FTIR) spectroscopy through comparison between two identical fabric samples, one of which is untreated and the other is treated. Our FTIR spectrometer (Bruker; model Vertex 80V) was operated in attenuated total reflection (ATR) sampling mode using a diamond anvil to explore the fabric’s surface characteristics. The transmittance measured in ATR units was obtained as a function of the input energy supplied in wavenumber units (${\text{cm}}^{-1}$).

**Figure 7.**
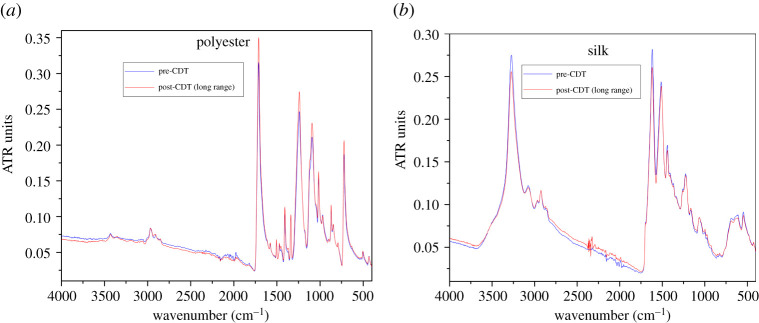
FTIR for (*a*) polyester and (*b*) silk fabric samples pre- and post-CDT at long range (5 cm).Data for this figure are available at [56]. (Online version in colour.)

Figure 6*b* presents a representative FTIR result for cotton fabric samples without (blue curve) and with CDT (red curve) performed at 3 cm distance between the discharge glow and the fabric sample. The most perceptible difference is that, at wavenumber $1728.19\;{\text{cm}}^{-1}$, the peak for the post-CDT sample is significantly higher than that for the pre-CDT sample; also, minor peaks in the 1158–$1278\;{\text{cm}}^{-1}$ range are suppressed in the untreated sample. These particular differences in the specific case of cotton are known to coincide with the presence of a (C=O) band, as reported at $1720\;{\text{cm}}^{-1}$ in [60] and at $1740\;{\text{cm}}^{-1}$ in [61], and a (C–O) functional group in the 1080–$1300\;{\text{cm}}^{-1}$ range [60]. In the process of looking for potential pitfalls in CDT-based electrocharging of knitted cloth fabrics, we in fact inadvertently confirmed the FTIR results in [60,61] for cotton fabrics exposed to low-temperature oxygen plasma. This reactive functionalization is not observed when the discharge is more than 3 cm from the fabric surface. Rather than cotton, in figure 7, we present pre- and post-CDT results for samples of knitted polyester (figure 7*a*) and silk (figure 7*b*) with the distance between the sample and the discharge set at 5 cm. The two results for pre-CDT (blue curves) and post-CDT (red curves) are nearly identical; in particular, no new peaks are observed.

Non-woven polymer fabrics are also known to undergo surface modification through reaction with active ionic species [62–65], with improvement of their adhesive properties being one of the better known applications [59]. In particular Shahidi *et al.* [65] reported the reactive surface treatment for polypropylene, the same material used in most FRs and SMs. However, we did not observe any such readings in FTIR measurements performed on SM samples and the reason for this is quite simple. All the aforementioned studies expressly applied either cold or low-temperature discharges, so none of their samples degraded under heat as our samples did. As presented above, our SM samples melt and structurally degrade under discharge heat at distances of up to 7 cm between the fabric and discharge. Any surface modifications on such fabrics is perhaps lost because of melting, but the implications themselves become moot. As we describe below, non-woven polymeric fabrics are electrocharged through oriented dipoles under CDT, whereas knitted cloth fabrics are perhaps electrocharged through adsorption of either electrons or free radicals on their surface, which has implications for charge retention and filtration efficiency as we discuss later.

Our tests on knitted cloth fabrics with the discharge–fabric vertical gap varied in steps of 1 cm each revealed an optimal working distance. As explained above, at short distances fabrics degrade structurally when the discharge glow and sparks come into contact with the fabric. At vertical distances above 5 cm we did not measure any charges. In an intermediate range of 3–5 cm discharge–fabric vertical distance we were able to measure the presence of charges on the fabrics, but this measured charge exhibited decay in time that varied over a wide range with fabric type. Whereas some fabrics did not get charged or discharged almost instantaneously, e.g. cotton, linen and silk, others exhibited a very long time decay over hours, e.g. polyester and cellulose. With no *a priori* expectation for CDT charging of knitted cloth fabrics, the discovery of charge retention in at least some cloth fabrics was an encouraging sign.

### (b) Charge measurements

#### (i) Corona discharge treatment and charge measurement protocol

We performed all electrostatic charge measurements with a non-contact electrostatic potentiometer (Kasuga Denki Inc.; KSS-2000 Digital Electrostatic Potentiometer) with a piezoelectric transducer capable of measuring electrostatic voltage at a distance of 50–100 nm from the sample. The potentiometer was connected to a laptop via a USB interface for automated data acquisition. During preliminary measurements, we noticed a confounding drift towards higher values in potentiometer readings after the first few minutes, which we isolated to charge accumulation on the sensor from prolonged exposure. This measurement anomaly was rectified by resetting the potentiometer before each measurement, which resulted in more reliable measurements; all measurements we report here follow this protocol.

Following our surface characterization studies, all non-woven polymer samples were treated at an electrode–sample gap of 8 cm, whereas knitted cloth samples were treated at 4 cm. This was also determined to be a safe distance at which the FM and FR samples did not suffer dimensional changes, especially from shrinkage, which could potentially lead to misfitting FMs and FRs. Whereas this was never a concern with knitted-cloth fabrics, we did observe localized wrinkles in polymeric samples due to local melting when the sample–electrode gap was less than 7 cm. The CDT system was operated above its breakdown voltage ($V_{B}∼10.5\;\text{kV}$) at 12 kV for improved ion flux at these operating distances, with the inductive ballast regulating current to attain its saturation value. There was sufficient ion flux to operate the linear guide rail holding the electrodes at $1\;{\text{cm s}}^{-1}$ translation speed for charge saturation in fabrics. Fabrics which we concluded were incapable of charge retention were exposed to CDT for as long as 10 min with no measurable charge retention. We measured neither their ionic presence nor their flux directly; instead, we exposed the electrostatic potentiometer’s sensor to the incident transverse electric wind, which registered a charge of −32 nC at a sensor–discharge vertical gap of 10 cm. We therefore concluded that there was more than sufficient ionic charge available to be imparted even at operating distances beyond the ones we selected.

#### (ii) Charge measurement results

**Figure 8.**
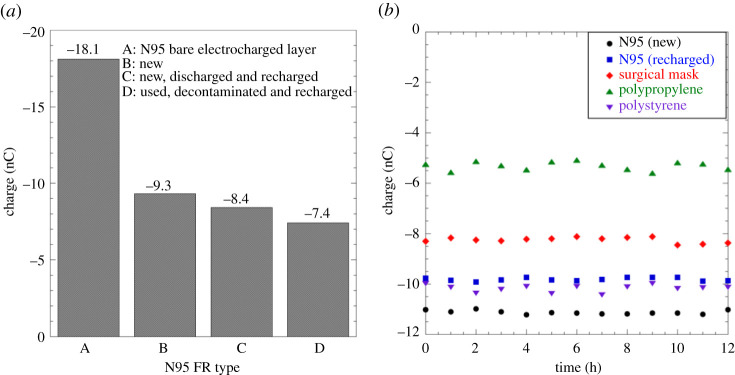
(*a*) Charge in nano-coulombs (nC) measured on N95 FRs under different conditions: bare N95 electrocharged layer (type A), new commercial N95 FR (type B), new, discharged and then recharged N95 FR (type C), and a used, decontaminated and recharged N95 FR (type D).(*b*) Charge as a function of time for new N95 (black solid circles), recharged N95 (solid blue squares), newly charged SM (red solid diamonds), polypropylene (upright solid green triangles) and polystyrene (inverted solid purple triangles) fabrics produced in-house exhibit steady charge readings over a 12 h duration. Data for this figure are available at [56]. (Online version in colour.)

N95 FRs with electrocharged filtration layers form the baseline comparative standard for all other fabrics. Our results therefore begin with charge measurements performed on N95 FRs and are detailed in figure 8*a* for charge (in nC) obtained from a single sample. Given that the N95 FR’s electrocharged layer lies sandwiched between outer non-electrocharged fabrics, charge measurement on the N95 FR’s outer surface is bound to be lower than the one measured directly on the electrocharged layer because of distance separation as well as the outer layer’s presence as a dielectric barrier. Indeed, the charge recorded for a bare electrocharged layer (type A in figure 8*a*) was −18.1 nC, nearly twice the value recorded at a new N95 FR’s outer surface (type B in figure 8*a*) at −9.3 nC. Post measurement, this new N95 FR was depleted of its charge by soaking it in ethanol, dried and then exposed to a balanced ion generator (CDT in bipolar mode). After confirming the presence of no net charge, this N95 FR was recharged using CDT to simulate a used, decontaminated and recharged FR (type C in figure 8*a*) and it recorded −8.4 nC. From this baseline test, we learned that recharging comes close to, but not quite, restoring N95 FRs back to their pristine charge values. As a control, a used and decontaminated N95 FR was also recharged via CDT protocol (type D in figure 8*a*) and it recorded −7.4 nC of charge. We speculate that the discrepancy of −1 nC between the type C and type D N95 FRs may be due to trapped particles and bioaerosols screening the charge in type D.

Polymeric electret fabrics are charged through oriented dipoles within the polymeric material and ideally retain their charge for as long as the electret fabrics are properly packaged and stored. Whereas we expect the same for CDT-recharged N95 FRs, it is not guaranteed; this requires testing because the instantaneous measurement in figure 8*a* does not inform us of charge decay and asymptotic steady charge values, if any. Readings were therefore obtained for commercial electret fabrics, both recharged (N95 FRs) and charged (SMs) for the first time. Charges recorded at 1 hour intervals for a 12 h duration (figure 8*b*) did not reveal any discharging: nearly steady charge was observed for a new N95 FR (black solid circles) with −9.3 nC mean value, intentionally depleted and CDT-recharged N95 FR (solid blue squares) with −8.4 nC mean charge and a new SM CDT charged for the first time (solid red diamonds) with −4.2 nC mean value. As a further test, non-woven polypropylene (upright solid green triangles) and polystyrene (inverted solid purple triangles) manufactured using the cotton candy principle detailed in [16] and subjected to the CDT process recorded steady values of −5.3 and −10.1 nC, respectively. All these samples when wrapped in Mylar plastic film and stored for four weeks still retained the same charge levels. We were therefore assured that these electret fabrics were charged by orienting the dipoles embedded in the polymeric material.

**Figure 9.**
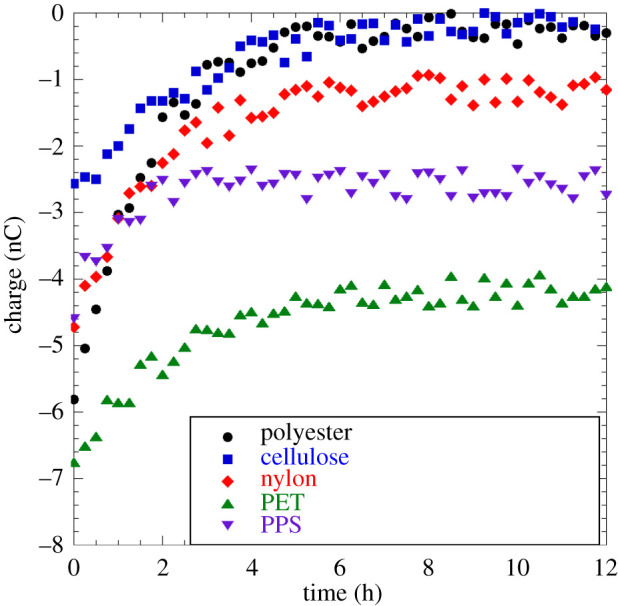
Charge as a function of time for knitted cloth fabrics: polyester (solid black circles), cellulose (solid blue squares), nylon (solid red diamonds),PET (solid green upright triangles) and PPS (solid purple inverted triangles). Data for this figure are available at [56]. (Online version in colour.)

Triboelectric charging is the only route by which knitted cloth fabrics are known to gain charge, a technique known since antiquity but whose fundamental mechanisms are only just beginning to be elucidated [33,66–68]. Applying the CDT method to knitted cloth samples, only a few and not all fabrics possess the ability to take up charges. Among those fabrics that we were able to charge, we found wide variability in their ability to gain charges in the first place as well as retain them. Unfortunately, some of the fabrics most commonly employed in FMs such as cotton, silk and linen did not take up charges under any circumstances we explored, including CDT for up to 10 min. Some fabrics, both natural (natural biodegradable polyester and cellulose) and synthetic (nylon, polyethylene terephthalate (PET) and polyphenylene sulfide (PPS)) were able to take up charges under CDT. Figure 9 shows charge measurements at 15 min intervals for knitted cloth samples over a 12 h duration. All knitted fabrics (natural or synthetic) exhibit an early peak charge value that relaxes over a wide time period to a steady value, which also varies widely. All peak charge values (in nC), decay times (in hours) and the asymptotic steady values (in nC) are tabulated in table 1.

**Table 1.** Charge measurements on knitted cloth fabrics.

fabric	peak charge (nC)	decay time (h)	steady value (nC)
cellulose (figure 9)	−2.6	6	0
polyester (figure 9)	−5.8	5.5	0
cotton	0	0	0
silk	0	0	0
linen	0	0	0
nylon (figure 9)	−4.7	3.5	−1.1
PET (figure 9)	−6.9	7	−4.2
PPS (figure 9)	−4.5	1.5	−2.6

Whereas non-woven polymer electret fabrics exhibit steady charge retention (figure 8*b*) from orientation of embedded dipoles, knitted cloth samples exhibit an initial peak charge that relaxes over hours to an asymptotic steady value that can be as low as 0 nC (figure 9). This points to a fundamentally different charge uptake mechanism in knitted cloth. Polymer electret cloth fabrics such as PET and PPS which asymptote to a reasonably high charge value of −4.2 and −2.6 nC, respectively, still exhibit early time relaxation from a peak value, the high non-zero asymptotic charge value most likely arising from orientation of dipoles. These observations taken together indicate the possibility of anion adsorption onto the fabric surface in knitted cloth and the slow discharging as a desorption mechanism.

### (c) Ion adsorption in knitted cloth fabrics

Experimental proof of adsorption involves adsorption isotherms where the adsorbate fraction is quantified against a range of external applied pressures at a fixed temperature; several isotherms at different temperatures chart the parameter space [69]. The functional form of these isotherms is derived from the virial equation and requires computational support for estimation of virial coefficients [70], failing which empirical adsorption isotherms [71,72] from experiment then inform theory. Several constraints prevent us from achieving complete characterization, which must await future study. We cannot measure ion concentration directly. Since air is a gaseous mixture [73] and not single component, measured charge is not a simple proxy for adsorbate concentration. For this same reason, simple empirical fits, e.g. Langmuir [71] or Freundlich [72] isotherms, do not automatically apply in our case and a fit to experimental data becomes pointless. Also, the virial coefficients cannot be estimated without computational support owing to the multi-component ionic composition. Furthermore, we did not have the necessary equipment to achieve pressure p>1 atm (∼100 kPa), but we could achieve lower pressures (p<1 atm).

In addition to all the above considerations which concern the adsorbate alone, adsorbent characteristics are also important [74]. Knitted fabrics possess a wide range of surface textures, porosity, material composition and chemistry, and chemical compositions arising from fabric treatment prior to use. All these taken together add an astonishing range of complexity to the characterization process that is beyond the scope of this proof-of-concept study. An illustrative example is provided by a comparison of polyester and PET. Whereas the polyester fabric we used was spun from natural, biodegradable yarn it is chemically close to PET. Indeed, PET often forms the base material for synthetic polyester yarns. Yet the two fabrics display markedly different charging behaviour. Whereas the natural, biodegradable polyester fabric had a high initial peak charge value, it completely discharges to 0 nC over 5.5 h. On the other hand, knitted fabric spun from PET yarn possessed a marginally higher initial peak charge relative to polyester and still retained the highest asymptotic charge value after 12 h.

The above constraints notwithstanding, electrocharging of knitted fabrics through ion adsorption is still provable, and is provided in two stages. To accomplish this, the experimental set-up was modified by enclosing the electrode system and fabric sample within a sealed bell jar. A vacuum pump connected to the bell jar was employed to evacuate the air within, and a valve together with a pressure gauge was used to control the pressure. The system’s temperature control is less straightforward since the corona discharge results in thermal energy release. The fabric sample was placed on a Peltier heating plate to control its temperature. The temperature was held fixed while the pressure was stabilized to the desired value followed by CDT on the sample and charge measurement.

**Figure 10.**
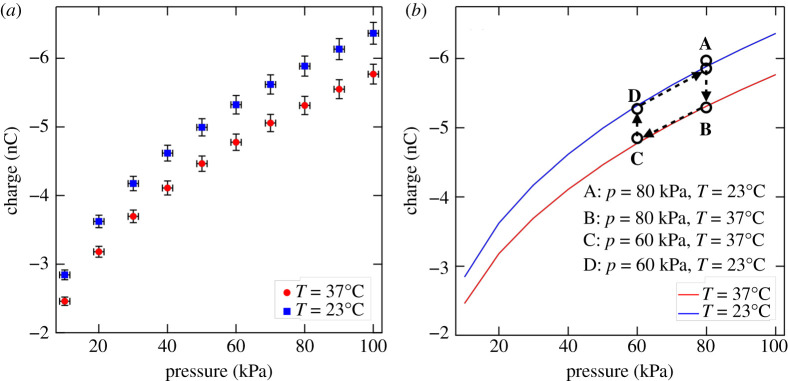
(*a*) Charge (nC) plotted against pressure (kPa) for a polyester sample at temperatures of $T=37^{\circ }\text{C}$ (red solid circles) and $T=23^{\circ }\text{C}$ (blue solid squares) establishes the dependence of net charge on the thermodynamic variables.(*b*) Evolving the system through states A–D completes a closed thermodynamic cycle through adsorption and desorption when either the pressure or temperature is varied while keeping the other fixed. Red ($T=37^{\circ }\text{C}$) and blue ($T=23^{\circ }\text{C}$) curves are experimental data plotted in (*a*). (Online version in colour.)

The first stage of the proof is presented in figure 10*a* with measurements performed on polyester fabric as a representative sample. Two isotherms at temperatures of $T=37^{\circ }\text{C}$ (red solid circles) and $T=23^{\circ }\text{C}$ (blue solid squares) for pressures in the range p=10–100 kPa in steps of 10 kPa are presented. The total charge measured at the fabric surface increases systematically as the pressure rises in accordance with an adsorption mechanism. The total charge is proportional to the total mass of the multi-component ionic species, hence it serves as a non-trivial proxy of unknown functional relationship for the adsorbate. Next, the isotherm shifts to higher charge values when the temperature is decreased from 37°C to 23°C; this behaviour also accords with an adsorption mechanism.

In the second test, we evolved the system through a thermodynamic cycle to test for the existence of phase equilibrium. Figure 10*b* shows the system evolve through states A–D (black open circles in figure 10*b*); see the legend in figure 10*b* for the thermodynamic variable values for each state. Solid red ($T=23^{\circ }\text{C}$) and blue ($T=37^{\circ }\text{C}$) curves are experimental data from figure 10*a*. Evolving the system from state A to state B, we notice charge desorption until it attains a value expected from the isotherm for $T=23^{\circ }\text{C}$. Further charge desorption occurs in evolving the system from state B to state C along the same isotherm as the pressure is reduced while temperature remains fixed. Next, the system adsorbs ions and increases its net charge in evolving from state C to state D (pressure held constant with a decrease in temperature) and cycles back to state A to within experimental error in the final step, thereby establishing phase equilibrium between the adsorption and desorption processes.

### (d) Filtration efficiency

Filtration efficiency tests were performed on all samples using a set-up constructed in-house. This filtration test set-up is described in full detail in [16], including the design shortcomings, which do not conform to some aspects of the N95 standard mandated by the National Institute for Operational Safety and Health (NIOSH). In brief, the set-up consisted of a manikin head placed in a confined box. An inlet into the box provided aerosolized sodium chloride droplets. A hole was drilled from the back of the manikin head to its mouth, where the test FM or FR was mounted, and a vacuum pump connected to the back of the manikin head provided suction. Two PM2.5 monitors were used to record aerosol particle concentrations: monitor A was placed in the confining box for input aerosol concentration and monitor B was placed between the manikin head and the vacuum pump to record the particle concentrations that were able to pass through the FR or FM.

All filtration tests were conducted under the following prevailing conditions. The laboratory temperature was maintained at $23\pm 2^{\circ }\text{C}$ with a relative humidity of 43%. The relative humidity within the filtration test set-up’s confining box was however higher owing to the presence of the aerosol at 58%. The area of the fabric samples used for FMs was 9 × 16 cm. All filtration tests were performed at 30 l m^−1^ flow rate and the aerosol particle concentration was $1.7\times 10^{8}\;\text{particles per}\;{\text{cm}}^{3}$, providing a high enough aerosol concentration.

Filtration efficiency is usually measured in terms of the penetration percentage (*P*), which is defined as the percentage of particles present in the environment that pass through the FRs and is quoted against the particle diameters. Since particle sizes could not be measured by the PM2.5 monitors we employed, the penetration is instead defined as:

4.1 $$P(t)=\frac{C_{B}(t)}{C_{A}(t)}*100\text{\%},$$

where $C_{A}(t)$ and $C_{B}(t)$ are the particle concentrations of PM2.5 monitors A and B, respectively, at time t. The penetration as a function of time was followed to study any deterioration in filtration properties. The PM2.5 monitors A and B were connected to a laptop and programmed to record concentration values at 15 min intervals over a duration of 12 h. Under this measure, filtration efficiency is 100% – P(t), i.e. the NIOSH-mandated N95 standard conforming to 95% filtration efficiency is equivalent to 5% or less penetration.

Commercial N95 FRs and SMs were used in pristine condition for the tests, whereas cloth masks were prepared from constituent fabric material with three layers. CDT was performed only on the outer layer exposed to the environment for all FRs and FMs, not the inner layer that comes into contact with the face. All FRs and FMs were taped to the manikin head to negate any possibility of air leakage through an interstitial gap between the mask and manikin face, as shown by prior results [16]. Filtration tests performed included one with a new commercial N95 FR to serve as the standard against which to measure all results. A second test was performed with N95 FR that was intentionally discharged (ethanol soaked, dried and exposed to a balanced ion generator) followed by CDT recharging. All filtration tests on surgical and knitted cloth FMs were performed in the natural uncharged (control) test we refer to as pre-CDT, followed by post-CDT charging to obtain a comparison with the same material. We will also present filtration test results for N95 FRs and SMs separate from knitted cloth FMs owing to the different charging mechanisms.

**Figure 11.**
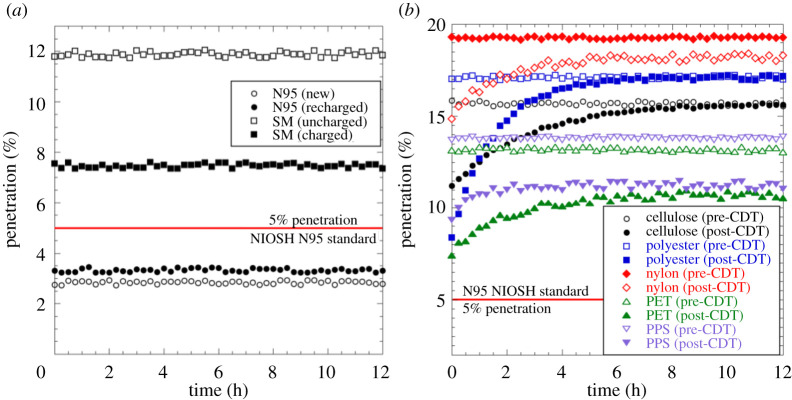
(*a*) Penetration (%) versus time (h) over a 12 h duration for a new commercial N95 FR (black unfilled circles), intentionally discharged and then CDT recharged N95 FR (black solid circles), a new commercial SM (black unfilled squares) and a post-CDT SM (black solid squares). The NIOSH-mandated N95 standard’s penetration value is shown as a solid red line at the 5% mark.(*b*) Penetration (%) versus time (hours) for knitted cloth FMs pre- (open symbols) and post-CDT (filled symbols): cellulose (black circles), polyester (blue squares), nylon (red diamonds), PET (green upright triangles) and PPS (purple inverted triangles). Data for this figure are available at [56]. (Online version in colour.)

Figure 11*a* presents filtration test results for N95 FRs and SMs from non-woven polymer electret fabrics, which exhibited the isothermal charging route to electrocharging. The 5% penetration value for the NIOSH-mandated N95 standard is marked as a red line, implying that all data that fall below this red line conform to the N95 standard, and all data above do not. As expected, a new (previously unused) commercial N95 FR as well as a new commercial N95 FR intentionally discharged via ethanol soaking, drying and exposure to a balanced ion generator and then recharged by CDT performed within the N95 standard. A standard SM pre-CDT yielded a penetration value of around 11.8%, well above the N95 standard, as expected for two simple reasons. SMs do not come pre-electrocharged, nor do they possess enough layers to provide the N95 standard protection. A tight facial fit is another important difference between N95 FRs and SMs, but that reason is rendered moot here as we intentionally taped all FRs and FMs to the manikin head in our tests. The correct comparison therefore is between pre-CDT and post-CDT SMs. When CDT charged, the same SMs performed much better with a penetration value of 7.45%. Whereas this value still falls short of the N95 standard, it is a considerable improvement over an uncharged SM.

Figure 11*b* shows filtration test results (penetration versus time) pre- and post-CDT for knitted cloth FMs, which exhibit the adsorption route to electrocharging. Two observations are immediately apparent from the results. Firstly, whereas penetration values are steady (time-independent) for pre-CDT cloth FMs, post-CDT they show a marked reduction in penetration (i.e. improvement in filtration efficiency) owing to electrocharging. Although the penetration values do not meet the N95 standard, they would offer improved filtration protection to the larger populace that relies on cloth FMs for protection. These initial penetration values relax over hours and track the discharging of the fabrics as observed in figure 9. Secondly, whereas cellulose and polyester penetration values converge to an asymptotic value that matches their pre-CDT levels, nylon, PET and PPS FMs relax to a lower penetration value, implying improved filtration efficiency even after the charges have desorbed from fabric surfaces. This observation is in perfect accord with results tabulated in table 1, where nylon, PET and PPS are the only fabrics that relax to non-zero negative charge values. This behaviour can only be attributed to the fact that there must be embedded dipoles in these three fabrics’ constituents that are oriented to a limited extent during CDT. Whereas the adsorbed ions are lost over time through desorption, the embedded dipoles within the fabrics are sufficiently oriented, much like electret fabrics, to impose an electric field that improves filtration efficiency. Note that we paid no attention to yarn thickness, fabric pre-treatment methods, etc., which certainly impact penetration values. For instance, the nylon fabric we employed in tests was quite coarse, which explains its high pre-CDT penetration percentage of nearly 19%. Our aim here was to explore charge uptake ability for knitted cloth fabrics; its reliance on material parameters must await future investigations. Penetration percentage values for all FRs and FMs are tabulated in table 2.

**Table 2.** Penetration values for tested FRs and FMs.

fabric	min. penetration (%)	decay time (h)	steady penetration (%)
N95 (new) (figure 11*a*)	2.84	0	2.84
N95 (recharged) (figure 11*a*)	3.32	0	3.32
SM (pre-CDT) (figure 11*a*)	11.88	0	11.88
SM (post-CDT) (figure 11*a*)	7.46	0	7.46
cellulose (pre-CDT) (figure 11*b*)	15.69	0	15.7
cellulose (post-CDT) (figure 11*b*)	11.23	6.75	15.7
polyester (pre-CDT) (figure 11*b*)	17.1	0	17.1
polyester (post-CDT) (figure 11*b*)	8.36	6	17.28
nylon (pre-CDT) (figure 11*b*)	19.26	0	19.26
nylon (post-CDT) (figure 11*b*)	14.86	4.25	18.4
PET (pre-CDT) (figure 11*b*)	13.21	0	13.21
PET (post-CDT) (figure 11*b*)	7.39	7	10.98
PPS (pre-CDT) (figure 11*b*)	13.79	0	13.79
PPS (post-CDT) (figure 11*b*)	9.32	2	11.1

## 5. Discussion

Having demonstrated CDT’s ability to electrocharge electret and knitted cloth fabrics via isothermal charging and ion adsorption, respectively, a few qualifiers are in order. Figure 10*b* informs us that charge depletion should occur more quickly in real-world conditions because ion desorption is accelerated by the human body’s elevated temperature relative to its surroundings. Furthermore, the presence of vapour in human breath increases relative humidity and would only act to further amplify charge depletion. We are unfortunately unable to quantify the charge depletion when such masks are worn by humans, and can only state that the decay time and penetration values presented in tables 1 and 2 are upper bounds. Secondly, the dependence of ion adsorption on thermodynamic variables points to the possibility that commonly used fabrics such as cotton or silk may adsorb charges at higher applied pressures or lower temperatures; this possibility too has not been explored since we lacked access to high-pressure equipment. The high diversity of fabric types and their surface finish notwithstanding, we investigated as many fabrics as we could commercially source to test for electrocharging capability under CDT; all fabrics had to meet the criterion that they were breathable. Table 3 lists all the fabrics that we investigated beyond those listed in tables 1 and 2.

**Table 3.** Expanded set of charge and filtration measurements on knitted cloth fabrics. *Note*. C, charge; P, penetration; PVC, poly vinyl chloride; PTFE, polytetrafluoroethylene; PDMS, poly dimethyl siloxane.

fabric	peak C (nC)	steady C (nC)	decay time (h)	min. P (%)	steady P (%)
polycotton	−0$.1$	$0$	$0$	$0$	$0$
Viton	−21$.2$	−20$.6$	$0$$.3$	$3$$.73$	$3$$.8$
polyacrylonitrile	−6$.3$	−6$.3$	$0$	$4$$.2$	$4$$.2$
acetal	−16$.6$	−16$.6$	$0$	$4$$.12$	$4$$.12$
Kevlar	−1$.4$	$0$	$1$$.2$	$9$$.46$	$0$
Garolite G-10	−18$.1$	−18$.1$	$0$	$3$$.22$	$3$$.22$
polysulfone	−4$.6$	−1$.9$	$3$$.25$	$8$$.6$	$10$$.2$
PVC	−8$.3$	−8$.3$	$0$	$6$$.3$	$8$$.1$
spandex	−2$.6$	$0$	$3$$.6$	$7$$.2$	$9$$.3$
PTFE	−11$.4$	−11$.4$	$0$	$3$$.4$	$3$$.4$
wool	$0$	$0$	$0$	$12$$.8$	$12$$.8$
polyurethane	−4$.8$	−3$.6$	$2$$.0$	$5$$.8$	$6$$.4$
polycarbonate	−6$.1$	−5$.6$	$5$$.75$	$4$$.6$	$5$$.3$
polyetherimide	−3$.5$	$0$	$2$$.4$	$4$$.4$	$7$$.6$
PDMS	−4$.6$	−2$.7$	$4$$.5$	$6$$.2$	$9$$.5$
Santoprene	−3$.6$	$0$	$3$$.2$	$5$$.4$	$8$$.1$

Table 3 extends the range of fabrics one might attempt to electrocharge through the CDT method. Do note that, since all fabrics were commercially sourced, we make the implicit assumption that they have been certified by a competent authority for safe use; we have not undertaken such an investigation ourselves. A more systematic exploration of the dependence of electrocharging ability and magnitude on fabric type, knit, pre-treatment, chemical composition, etc. under CDT must await further investigation. It is our earnest hope to undertake such an investigation and compile a CDT electrocharging series similar to the triboelectric series in future. This is of particular interest given that tribolelectric charging is the only previously known method for imparting charges to fabrics. Comparing these two methods, triboelectric charging suffers from strong protocol dependence which CDT does not. This protocol dependence imparts huge variability to charges imparted to fabrics by triboelectric charging; this is not so with CDT, which gives nearly identical results to within experimental error under identical thermodynamic conditions. However, CDT is relatively more expensive to set up than triboelectric charging, and in the same vein triboelectric charging is also a technically simpler solution than CDT.

The same health and safety considerations apply to both triboelectric and CDT methods, which come under the British Standard BS EN 7506-1:1995 [75]. This Standard is couched in terms of a threshold voltage of 3.6 kV, above which a human body elicits a physiological response, i.e. if the body voltage is 3.6 kV it is sufficient to cause a perceptible reaction level in a human being. A value higher than this causes problems, including sensation at around 11.5 kV and even an unpleasant shock at 26.5 kV. This issue does not arise in our CDT protocol for two reasons. Firstly, the charge density in most, but not all, fabrics is not high enough to breach the threshold voltage of 3.6 kV, and secondly we only impart charges on the outer surface of the FR or FM that is exposed to the environment and not to the inner surface that comes into contact with the skin. Even the adsorbed ions on the FR and FM outer surfaces do not possess a high enough concentration relative to air to cause any respiratory distress.

## 6. Conclusion

In summary, we have presented an experimental method to electrocharge N95 filtering FRs and FMs derived from non-woven polymer electret fabrics as well as natural or synthetic knitted cloth fabrics using CDT. In keeping with our focus, we presented two designs constructed from easily sourced components not subject to global logistics disruptions.

Our tests revealed that N95 FRs and SMs fabricated from non-woven polymer electret fabrics undergo electrocharging via the well-known isothermal charging route. Several, but not all, knitted cloth fabrics did exhibit electrocharging despite no *a priori* expectation. Charging in knitted cloth fabrics arose from ionic adsorption, a mechanism different from isothermal charging.

Filtration tests confirmed a behaviour similar to the charging mechanism observed in non-woven polymer electret and knitted cloth fabrics. New and reused N95 FRs as well as SMs exhibited steady penetration in line with constant charge over time. SMs exhibit a measurable improvement in filtration efficiency from electrocharging, but fall short of achieving the N95 filtration standard. We confirmed that cloth FMs that exhibit steady penetration values pre-CDT were able to reduce penetration post-CDT to an initial minimum value on the same cloth-based FM. The penetration values progressively increased in time and tracked the charge decay over a few hours depending upon the fabric type, before converging to a steady value. Whereas this steady value coincided with pre-CDT penetration values in some fabrics that completely discharged, it was lower than pre-CDT penetration values in other fabrics, in line with charge measurement results which showed a non-zero asymptotic charge value in these fabrics. All the above progress notwithstanding, a substantial amount of work remains, especially towards understanding CDT electrocharging in knitted cloth fabrics as detailed at appropriate junctures throughout the article.

We close with the earnest hope that this study will both prove useful in improved mask-based respiratory protection and also spur further research into simple electrocharged filtration strategies based on knitted cloth fabrics capable of mass deployment.

## Data accessibility

Data are available from the Dryad Digital Repository [56].
